# Ultrathin (<1 μm) Substrate-Free Flexible Photodetector on Quantum Dot-Nanocellulose Paper

**DOI:** 10.1038/srep43898

**Published:** 2017-03-07

**Authors:** Jingda Wu, Lih Y. Lin

**Affiliations:** 1 Department of Electrical Engineering, University of Washington, 185 Stevens Way, Seattle, WA 98195-2500, USA.

## Abstract

Conventional approaches to flexible optoelectronic devices typically require depositing the active materials on external substrates. This is mostly due to the weak bonding between individual molecules or nanocrystals in the active materials, which prevents sustaining a freestanding thin film. Herein we demonstrate an ultrathin freestanding ZnO quantum dot (QD) active layer with nanocellulose structuring, and its corresponding device fabrication method to achieve substrate-free flexible optoelectronic devices. The ultrathin ZnO QD-nanocellulose composite is obtained by hydrogel transfer printing and solvent-exchange processes to overcome the water capillary force which is detrimental to achieving freestanding thin films. We achieved an active nanocellulose paper with ~550 nm thickness, and >91% transparency in the visible wavelength range. The film retains the photoconductive and photoluminescent properties of ZnO QDs and is applied towards substrate-free Schottky photodetector applications. The device has an overall thickness of ~670 nm, which is the thinnest freestanding optoelectronic device to date, to the best of our knowledge, and functions as a self-powered visible-blind ultraviolet photodetector. This platform can be readily applied to other nano materials as well as other optoelectronic device applications.

Recently, nanocellulose paper has emerged as a very promising transparent substrate material for flexible devices^1^,^2^,^3^ due to its superb mechanical and optical properties^3^, and has been applied to making LEDs^4^, displays^5^, transistor arrays^6^, solar cells^7^,^8^, foldable nanoantennas^9^ and touch screen coatings^10^ with high performance. Made from the same cellulose materials as regular paper but with new engineering methods to reduce the size of the cellulose fibers, the nanocellulose paper can achieve high transparency (>90%) and smoothness (<10 nm). Furthermore, nanocellulose is biodegradable and environmentally friendly, which is another advantage over glass, plastic or organic flexible materials^4^,^11^. With their high surface-to-volume ratios and surface functional groups, nanocellulose also offers room for functionalization. Researchers have immobilized various nanoparticles in nanocellulose networks such as Fe_3_O_4_ and CoFe_2_O_4_ nanoparticles to create magnetic nanopaper^12^,^13^, ZnSe QDs for making fluorescent paper^14^, and plasmonic metal nanoparticles for optical sensing^15^. However, the large thicknesses of these functional papers, over 30 μm for the Fe_3_O_4_ magnetic nanopaper^12^ and much higher for the others, places a significant challenge for using these papers as the active layers in optoelectronic devices, which require thin active layers to facilitate charge transport through the nanomaterials. Previously, flexible photodetectors have been demonstrated by embedding semiconductor QDs in the cellulose structures from natural plant membranes^16^. While photodetection function was achieved, the 5 μm membrane thickness is still too large for efficient charge transport. The irregular hierarchical structure of the membrane and large microfibril clusters also renders roughness on the membrane surface as well as makes QD distribution in the cellular network less uniform and lower concentrated. In this work, we demonstrate ultrathin freestanding QD films with nanocellulose structuring to achieve substrate-free flexible optoelectronics. Although QDs are utilized in our work, the approach can be extended to other solution-processable nanomaterials.

We first investigated the main challenges for fabricating ultrathin nanocellulose films. With its hydrophilic nature, nanocellulose is commonly dispersed in a hydro environment during fabrication, and the water capillary force plays a significant role in the processes. Common nanocellulose paper making processes consist of filtration, pressing and drying of excess water^12^. After filtration, the capillary force from the water and the cohesion force between nanocelluloses help the structure reach a hydrogel “cake” shape, which remains intact when it is being peeled off from the filter. However, when the hydrogel cake thickness is reduced to a couple of micrometers or less, the water capillary force between the hydrogel and the filter, which only depends on the interface between the filter and hydrogel and is independent of the hydrogel thickness, will be similar and eventually becomes stronger than the forces sustaining the gel, thus making it difficult to be peeled off. Furthermore, the strong water capillary force tends to warp the nanocellulose film, which could be detrimental to the ultrathin nanocellulose paper even if they can be peeled off from the filter. Therefore, to achieve freestanding ultrathin nanocellulose papers with high structure integrity, it is critical to find a way to overcome the water capillary force. Here we report a facile process to fabricate ultrathin (<1 μm) freestanding ZnO QD films with nanocellulose structuring by modifying conventional nanocellulose paper making process to incorporate a solvent-exchange step to mitigate the above-mentioned issue.

The nanocellulose solution is first prepared through a 2,2,6,6-tetramethylpiperidine-1-oxyl (TEMPO)-mediated oxidation process and high pressure microfluidizing^17^. The precursors for the active nanocellulose paper are made through mixing ZnO QDs in water and the nanocellulose solution (see Methods). Oxide nanocrystals have hydroxyl groups on the surface due to dangling oxygen bonds, which help them to suspend in water and interact with the carboxyl groups in nanocelluloses to form stable composites^12^. An ultrasonic homogenizer is used to homogenize the suspension during mixing to avoid regional excess ZnO QDs, which could lead to agglomeration and precipitation of ZnO QD-nanocellulose clusters. A ZnO QD to nanocellulose weight ratio of 0.75:1 is achieved through this process. The product is subsequently centrifuged to precipitate out bigger clusters due to agglomeration to form a colloidal suspension of homogeneous ZnO QD-nanocellulose composites, and the result is shown in Fig. 1. Translucent ZnO QD-nanocellulose suspension can be seen with good homogeneity (Fig. 1a). Bright yellowish photoluminescence (PL) can also be observed under 365 nm wavelength UV excitation (Fig. 1b). The PL properties of the QDs in the composites remain unchanged for a long period (no degradation was observed in a couple of months), while the untreated QDs tend to agglomerate in water and could lose their PL intensity in just several hours. This confirms the stability of the ZnO QDs in the composites. UV-vis absorption measurements reveal that ZnO QDs contribute to substantial light absorption at near UV range (Fig. 1c), which is essential for photodetector applications. Due to the wide bandgap of ZnO QDs (3.37 eV for bulk ZnO), no absorption is observed at visible range and this could potentially be utilized for “visible-blind” UV photodetection applications such that no filters are needed under broad illumination spectra if detection of visible wavelengths and beyond is undesirable.

The key steps in the ultrathin active nanocellulose paper-making process are depicted in Fig. 2. The QD-nanocellulose mixture solution first goes through vacuum filtration to form a thin “gel cake” on the filter (not shown in the figure), as in a typical nanocellulose paper fabrication process. To prevent the damage during peeling off the ultrathin nanocellulose paper at the final step due to water capillary force as described before, we first transfer the gel cake on a PVDF filter to an acrylic plastic plate (Fig. 2a, see Methods). After peeling off the filter, the entire gel cake/acrylic plate structure is soaked in isopropanol (IPA) for solvent exchange, which results in a free-floating gel cake in IPA (Fig. 2b). Acrylic was chosen as a preferred transfer substrate because IPA has much higher wettability on acrylic compared to other materials such as stainless steel (Supplementary Fig. S1) due to a high critical surface energy. Therefore, IPA can diffuse better between the gel cake and the acrylic plate, making it easier to separate the gel cake from the substrate. The freestanding QD-nanocellulose gel cake is subsequently pressed and dried to form the ultrathin active nanocellulose paper using a double-sided drying setup between two smooth PVDF filters (Fig. 2c, see Methods).

The ultrathin ZnO QD-nanocellulose paper fabricated using the process described above achieves very high transparency, as shown in Fig. 3a. It has higher transparency than a glass cover slide (~90% transparency at visible wavelengths) and significantly higher than tracing paper which is commonly noted for its transparency and has been utilized for making flexible devices^18^,^19^. Strong thin-film interference is observed visually when viewed at an angle (Supplementary Fig. S2) and in the UV-vis transmission spectrum (Fig. 3b), indicating high film quality in terms of smoothness. By analyzing the thin-film interference in the transmission spectrum, the thickness of the film is estimated to be ~528 nm (Supplementary Information). This is confirmed with scanning electron microscopy (SEM) characterization (Fig. 3c), which shows a thickness of ~550 nm. The surface of the QD-nanocellulose paper is very smooth with little roughness seen in the cross-sectional SEM image (Fig. 3c) as well as top-view SEM images (Fig. 3d and e) which show no fibrous networks. A thicker QD-nanocellulose paper was also fabricated (Supplementary Fig. S4). Although thicker, the paper has higher transparency (>95%) at visible range and exhibits weaker thin-film interference. A close look at the flat thin-film surface (Fig. 3e) reveals a structure consists of densely-packed QD-nanocellulose composites ~20 nm in size. The high concentration and uniform coverage of QDs is important for optoelectronic devices which require efficient carrier transport. The smooth surface is also essential for depositing additional high-quality films in device fabrications. Figure 3f shows the yellowish PL from the freestanding QD-nanocellulose paper under 365 nm UV light excitation. Similar PL spectra are obtained from the ultrathin ZnO QD-nanocellulose paper and composite suspension (Supplementary Fig. S5), confirming that the optical quality of ZnO QDs is not compromised during the active nanocellulose paper fabrication process.

Schottky junction photodetectors (Fig. 4) utilizing the QD-nanocellulose paper as the active layer with a vertical structure are fabricated through simple steps of thermal evaporation of metal electrodes on both sides of the active layer (Fig. 4a). A 100 nm-thick aluminum layer is used as the backside electrode and forms an Ohmic contact with the ZnO QDs. A 20 nm-thick gold layer is used as the semi-transparent electrode and forms a Schottky junction with the ZnO QDs (Fig. 4b). The sheet resistance of the gold electrode was measured to be <50 Ω/ϒ. Adding these layers, the overall device thickness is merely ~670 nm and is the thinnest freestanding flexible optoelectronic device that has been reported to date^20^,^21^,^22^,^23^, as far as we know. The device is ultra-flexible and lightweight, and can be easily attached to a curved surface through electrostatic force, as shown in Fig. 4c.

Current-voltage (I-V) characterization results show that the device exhibit rectifying diode behavior (Fig. 5a). When the device is under UV illumination, the built-in electric field in the Schottky junction helps separate the photo-generated carriers even under zero bias. Therefore, the device can function as a self-powered photodetector without external bias to save energy. The photoresponsivities of the device under different wavelengths were measured at zero bias for a wavelength range of 320–500 nm (Fig. 5b). It shows rapid increase in photoresponsivity as the wavelength decreases below 350 nm, in accordance to the UV-vis transmission spectrum of the ultrathin paper (Fig. 3b), and a peak response of ~3.65 mA/W is observed at ~343 nm. The noisier spectrum data at shorter wavelengths is mostly due to the low illumination power (Supplementary Fig. S7) which results in low photocurrent and larger error when converting to photoresponsivity. No response is observed at visible wavelength range due to a lack of absorption and the device can function as a “visible-blind” photodetector. Photoresponses of the device under different illumination powers are plotted in Fig. 5c as the optical power increases over time with off periods in between. The results show consistent behavior with good repeatability and device reliability. Given an illumination area of ~4 mm^2^, the photocurrent shows a linear dependence (Fig. 5d) on the illumination light power, indicating that the device is not saturated in this intensity range.

The performance of the device can be further improved by optimizing the device structure. One approach currently under investigation is to insert a thin layer of MoO_3_ between the QD-nanocellulose active layer and the gold electrode (Fig. 6a). Thin-film MoO_3_ is commonly used as a hole-transport layer for solar cells and LEDs. It has very high electron affinity and may potentially help carrier extraction^24^,^25^. The I-V characterization result of such a photodetector is shown in Fig. 6b. No current is observed at zero bias due to an additional Schottky junction introduced by the silver contact. Under 365 nm-wavelength illumination, the device achieved a photoresponsivity of ~9.6 mA/W at −0.5 V bias, which is roughly 40 times higher than the device without MoO_3_ layer at 0 V bias, note that the original device has similar responsivities under 0 V and −0.5 V bias because the main contribution of the current comes from the reverse saturation current.

In summary, we have demonstrated a facile approach to fabricating sub-micrometer, freestanding and transparent ZnO QD active layer with nanocellulose structuring. The fabrication process is enabled by hydrogel transfer printing and solvent exchange to overcome the water capillary force during the QD-nanocellulose paper making. The optical property of ZnO QDs is not compromised during the process, and ultrathin flexible Schottky photodiodes with a substrate-free device structure were realized using the QD-nanocellulose paper as the active layer. This fabrication process can be applied to other kinds of QDs or nanomaterials, with broad application potentials for optoelectronic devices involving photodetection and light emission. The fabrication approach is derived from paper-making process, and therefore can potentially be further developed into larger-scale manufacturing in the future.

## Methods

### ZnO Quantum Dot Synthesis

ZnO QDs are synthesized using a well-developed wet-chemistry method^26^,^27^. 2.95 g zinc acetate dihydrate is dissolved in 125 ml methanol. 1.48 g potassium hydroxide is dissolved in 65 ml methanol and mixed with the Zn(Ac)_2_ ⋅ 2H_2_O solution drop-wisely at ~65 °C. The reaction takes 2.5 hours with the solution turning turbid, followed by centrifuging to precipitate out the QDs. The precipitation is washed in methanol twice with centrifuging and then suspended in 20 ml methanol to form a clear and transparent solution with a ZnO QD concentration of ~34 mg/ml. The solution is stored at 4 °C and air-stable for months.

### Nanocellulose Preparation

Nanocellulose solutions are prepared according to the procedure reported in literature^12^,^17^ with a slight modification. 78 mg 2,2,6,6-tetramethylpiperidine-1-oxyl (TEMPO) is dissolved in 100 ml deionized (DI)-water and mixed with 514 mg NaBr after it has been dissolved in 50 ml DI-water. The mixture is added to 5 g dry bleached kraft pulp in 65 ml DI-water. After these, 30 ml ~12% NaClO is added to start the oxidation with strong magnetic stirring. The pH of this reaction is controlled to be ~10.5 by adding 0.5 M NaOH solution. After 2.5 hours, stop adding NaOH but continue stirring the mixture for another 2 hours or more for complete oxidation. Büchner filtration is used to drain all the solutions out of the end product, which is then washed with DI-water a couple of times until it becomes all white. Suspend the product in 500 ml DI-water to create 1 wt% dispersion of oxidized cellulose. At this stage the solution still contains micro-sized celluloses. Mechanical treatment is needed to further break the cellulose structure down to nanoscale. This can be achieved by running the solution through a microfluidizer (Microfluidics, Inc., M-110P) to fully deconstruct the cellulose structures into nanocelluloses. The nanocellulose solution is stored at 4 °C.

### ZnO QD and Nanocellulose Mixing

The above-synthesized ZnO QD suspension is diluted to a ZnO QD concentration of 1 mg/ml by adding DI-water. The nanocellulose solution is diluted to 0.1 wt% with DI-water and degassed for half hour before mixing. A volume ratio of 0.75:1 of ZnO QD and nanocellulose suspensions are mixed in a glass bottle and sonicated for 5 mins with an ultrasonic homogenizer (Biologics, Inc., Model 3000). The product is centrifuged under 2000 rpm for 10 mins to precipitate out larger clusters. The supernatant is then diluted again to half concentration by adding DI-water and the resulting solution can be readily used for active paper making.

### Ultrathin QD-Nanocellulose Paper Making and Device Fabrication

3 ml of the above mixture is filtered using a PVDF filter (Millipore, Inc.) with a pore size of 0.1 μm to form a thin wet “gel cake”. The gel cake on the PVDF filter is then immediately transferred to a clean acrylic plastic, and the filter is peeled off. We found that a better transfer result can be achieved by peeling off the filter with a quick movement, which is contradictory to the traditional transfer printing processes^28^. This could be related to the redistribution of water capillary force^29^,^30^ due to the hydrophilic and porous nature of the PVDF filter, which differs itself from an elastic smooth surface such as PDMS typically used in other transfer printing processes. Afterwards, the gel cake/acrylic structure is soaked in IPA for solvent exchange, and the gel cake can be separated from the acrylic with a little shake. The free-floating gel cake is spooned out with a PVDF filter and covered with another filter. The sandwiched structure is subsequently placed between two stacks of normal papers and fixed between two metal plates under pressure. The entire structure is left in a vacuum desiccator overnight for drying. All additional layers in the devices are deposited and patterned using thermal evaporation through shadow masks at a vacuum level <10^−6^ torr.

### Device and Material Characterization

**Figure 1:**
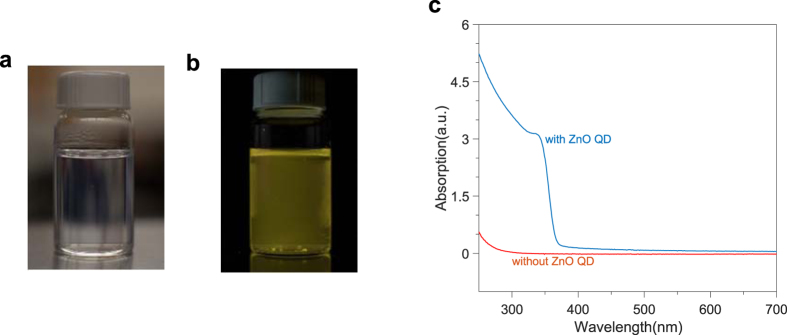
Ultrathin QD-nanocellulose paper precursor. Photos of ZnO QD-nanocellulose composites in water under room light (translucent) (**a**) and under 365 nm UV excitation (**b**). (**c**) UV-vis absorption measurement results of the nanocellulose thin films on glass slides with (blue curve) and without (red curve) ZnO QDs. The discrepancy between the two curves at longer wavelengths (>370 nm) is possibly due to light scattering, which comes from slightly increased inhomogeneous nanocellulose distribution on the glass slide when QDs are present.

**Figure 4:**
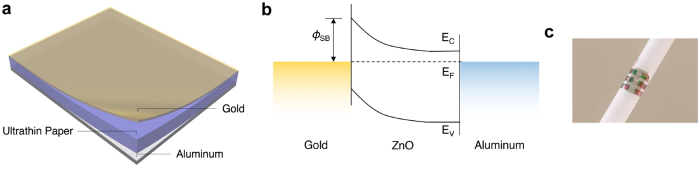
Substrate-free flexible UV photodetectors fabricated on the ultrathin QD-nanocellulose paper. (**a**) A schematic illustration of a Schottky photodiode structure for the substrate-free photodetector. The layers from bottom to top are aluminum back contact (grey), ultrathin QD-nanocellulose active layer (purple), and semi-transparent gold electrode (yellow). (**b**) A band diagram of the photodetector. (**c**) A photo of fabricated devices wrapped around a pen through electrostatic force. The greenish semi-transparent film is the gold electrode with a thickness of 20 nm, and 6 silver patterns are the aluminum back contacts, with a thickness of 100 nm.

Device responses are measured with a Keithley 6430 sourcemeter through NI LabView automation. A tungsten light source is used to illuminate the device through an Acton Research SpectraPro 275 monochromator to provide wavelength selectivity for the EQE measurement. 350 nm wavelength UV light is used for current-voltage, time response and saturation characterizations in Fig. 4d,f and g. A 365 nm UV LED is used for the study of the device with MoO_3_. The SEM images are obtained by a Sirion SEM. UV-vis absorption and transmission spectra are measured with Varian Cary 5000 UV-vis-NIR Spectrophotometer. The UV-vis transmission spectra in Fig. 1c was obtained by drop-casting materials onto clean glass slides and dried in the air. All measurements are performed at room temperature (300 K).

## Additional Information

**How to cite this article**: Wu, J. and Lin, L. Y. Ultrathin (<1 µm) Substrate-Free Flexible Photodetector on Quantum Dot-Nanocellulose Paper. *Sci. Rep.* **7**, 43898; doi: 10.1038/srep43898 (2017).

**Publisher's note:** Springer Nature remains neutral with regard to jurisdictional claims in published maps and institutional affiliations.

## Supplementary Material

Supplementary Information

## Figures and Tables

**Figure 2:**
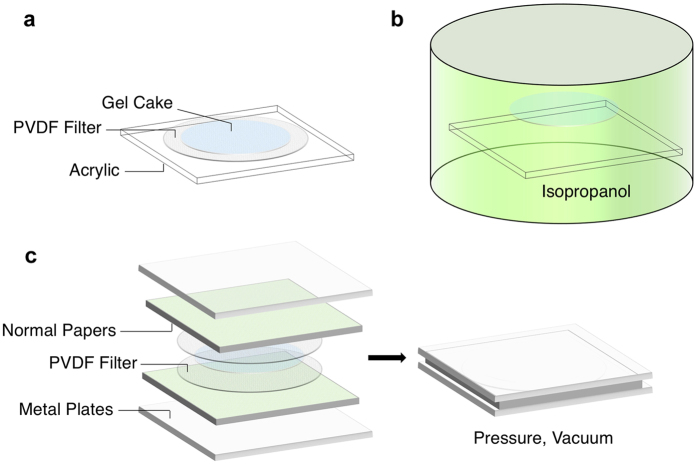
Schematics of the key steps in the ultrathin QD-nanocellulose film fabrication process. (**a**) After vacuum filtration, the thin wet QD-nanocellulose composite “gel cake” on a PVDF filter is transferred to an acrylic plastic plate and the filter is peeled off. (**b**) The gel cake/acrylic structure is soaked in IPA for solvent exchange and the gel cake floats freely in the solvent. (**c**) The floating gel cake is scooped out and sandwiched between two PVDF filters. This structure is then sandwiched between two stacks of flat porous materials (e.g., normal paper) to help the solvent evaporate. Pressure is applied to flatten and compress the films using two metal plates, and the entire setup is stored in a desiccator overnight for drying.

**Figure 3:**
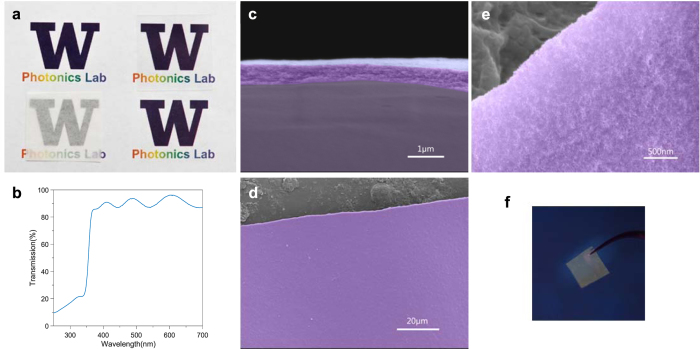
Fabrication results of ultrathin freestanding ZnO QD-nanocellulose paper. (**a**) The transparency of the ultrathin film (lower right) is compared with air (upper left), a glass cover slide (~90% transparency, upper right), and a piece of tracing paper (lower left). The active nanocellulose paper shows higher visual transparency than the cover slide and the tracing paper. (**b**) UV-vis transmission spectrum of the ultrathin QD-nanocellulose paper. An overall >91% transparency is observed with strong thin-film interference. The ZnO QDs result in strong UV absorption, as expected **c**, a false-color cross-sectional SEM image to show the sub-μm thickness (~550 nm) of the ultrathin QD-nanocellulose paper. The film is found to be ultra-smooth with little roughness seen in the cross-sectional image, which is also confirmed in the top-view SEM images (**d,e**). The purple region in the SEM images is the active thin film, which is found to consists of QD-nanocellulose composite elements ~20 nm in size. No fibrous networks are seen. The scale bars in (**c**,**d** and **e)** are 1 μm, 20 μm and 500 nm respectively. (**f**) Yellowish PL is observed under 365 nm wavelength UV light excitation.

**Figure 5:**
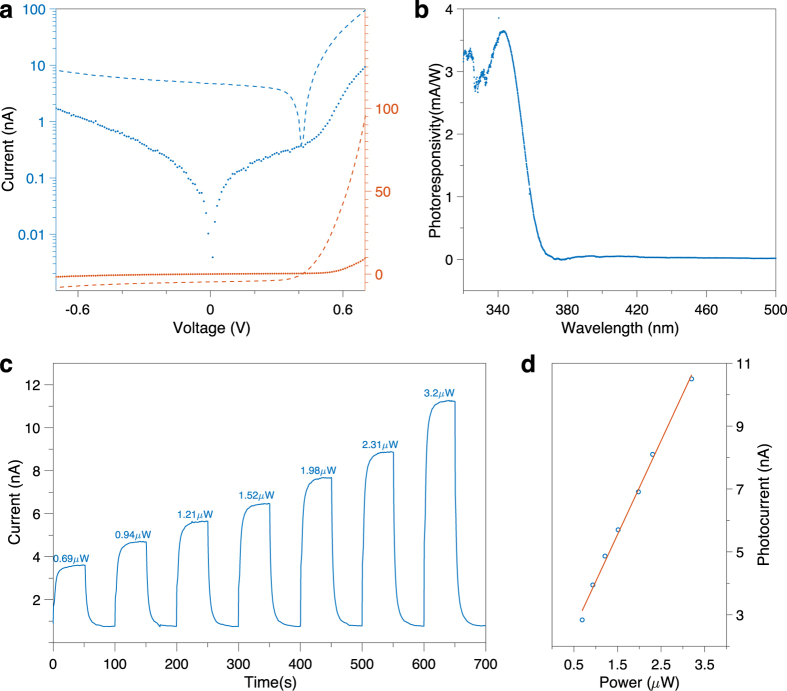
Performance of substrate-free flexible UV photodetectors. (**a**) Current-voltage (I-V) response of the device in log scale (blue) and linear scale (red), with (dash line) and without (dot line) UV illumination. (**b**) Photoresponsivities of the device at different wavelengths under zero bias. (**c**) Time responses of the device under different illumination powers. (**d**) Photocurrent versus illumination intensity, showing a linear response. The blue dots are experimental data and the red line is the linear fitting curve.

**Figure 6:**
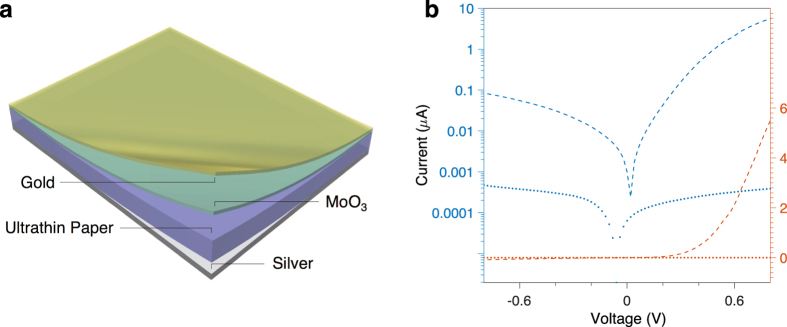
Substrate-free UV photodetectors with MoO_3_ layer insertion for carrier extraction. (**a**) A schematic illustration of a UV photodetector using a thin MoO_3_ layer to enhance carrier transport. The layers from bottom to top are silver back contact (100 nm, grey), ultrathin ZnO QD-nanocellulose film (purple), MoO_3_ (15 nm, green) and gold semi-transparent electrode (20 nm, yellow). (**b**) Current-Voltage response of the device in log scale (blue) and linear scale (red), with (dash line) and without (dot line) 365 nm UV illumination (4.4 μW).
