# Supplementary material for: Ultrathin (<1 μm) Substrate-Free Flexible Photodetector on Quantum Dot-Nanocellulose Paper
Source: Sci Rep. 2017 Mar 7;7:43898. doi: 10.1038/srep43898 (PMC5339685; doi:10.1038/srep43898)
Supplement: Supplementary Information [file srep43898-s1.pdf]

Ultrathin (<1 μm) Substrate-Free Flexible Photodetector on Quantum Dot-Nanocellulose Paper

Jingda Wu, Lih Y. Lin

1. Isopropanol (IPA) wettability

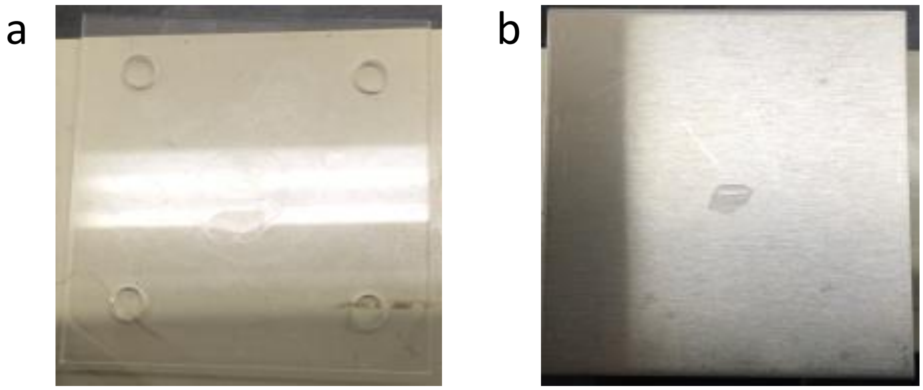

Figure S1 Comparison of the wettability of IPA on acrylic plastic and stainless steel. Photos of IPA droplets spreading on acrylic plastic (**a**) and stainless steel (**b**). A much larger wetting area is observed on acrylic.

2. Thin-film interference

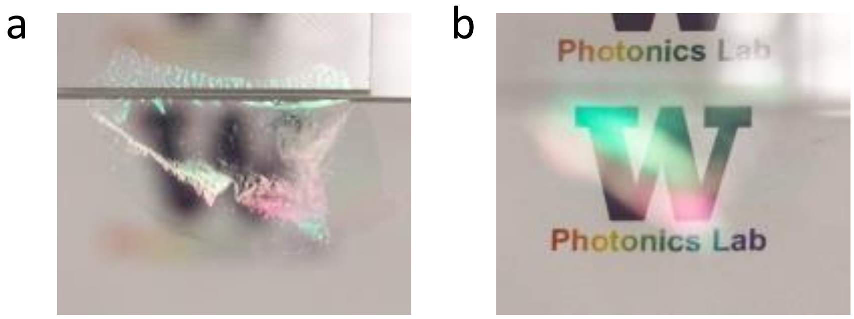

Figure S2 Photos of the ultrathin QD-nanocellulose paper showing high transparency and thin-film interference. Photos of the ultrathin film at a distance from a background image, taken with (**a**) focusing on the film and (**b**) focusing on the background image. The background image is clear in (**b**), indicating little to no scattering when visible light passes through the film. Thin-film interference is shown when the film is viewed at an angle, for example under bending.

Ultrathin film thickness calculation through thin-film interference analysis

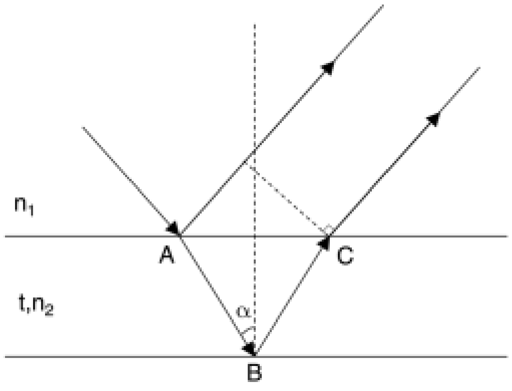

Figure S3 A schematic illustration of thin-film interference. n_1 and n_2 are refractive indices of the environment and the thin film, respectively. t is the thickness of the thin film and α is the reflection angle of the light in the thin film at the second interface.

Thin-film interference equation is given as[1]

$$2n_2 t\cos\alpha = m\lambda(m = 1, 2, 3...),$$

where n_2 and t are the refractive index and the thickness of the thin film, α is the reflection angle of the light in the thin film at the second interface and λ is the wavelength of the light. The environment is assumed to be air with $n_1 = 1$. From the UV-vis transmission data depicted in Figure 3b in the main text, the interference valleys obey the above equation and they occur at λ = 443nm ($n_2 = 2.105$), 542nm ($n_2 = 2.024$) and 689nm ($n_2 = 1.976$).With ZnO having a much larger refractive index ($n \simeq 2$) than does the cellulose ($n \simeq 1.5$), waveguiding effect could be formed with light propagating mostly in ZnO QDs. Therefore, we use the refractive index of ZnO as an estimation for n_2. The refractive indices for ZnO are 2.105, 2.024 and 1.976 at λ = 443nm, 542nm and 689nm. Since these interference valleys are adjacent to each other, they should correspond to integer m in series. The absorption spectrum was measured with light incident at 90°. A set of m satisfying the interference equations is m = 5, 4 and 3 for λ = 443nm, 542nm and 689nm, respectively. This corresponds to film thicknesses of 526.1nm, 535.6nm and 523.0nm. An average of 528nm is used as the estimation for the film thickness.

3. Results of a thicker QD-nanocellulose paper

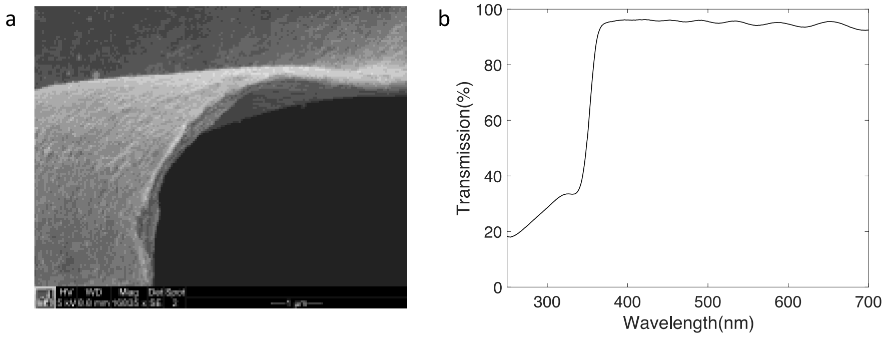

Figure S4 A thicker ZnO QD-nanocellulose paper. **a**, An SEM image of the film. **b**, UV-vis transmission spectrum showing that the film has a transparency >95% at visible range with less prominent thin-film interference.

4. PL results for the precursor and ultrathin paper

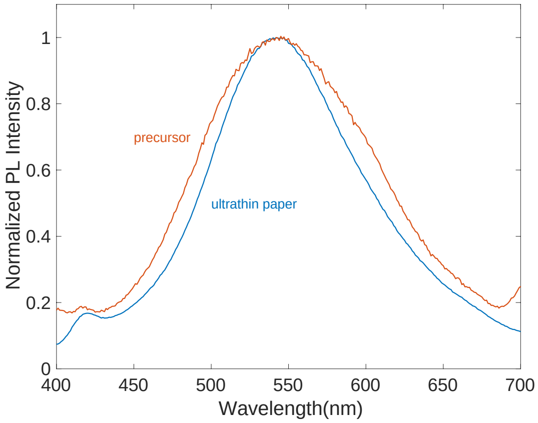

Figure S5 Photoluminescence (PL) measurement results of ZnO QD-nanocellulose in ultrathin paper and in suspension excited under 365nm UV light. The thin film (blue curve) shows a similar PL spectrum as the solution (red curve), confirming no degradation on the optical quality when the suspension is made into ultrathin QD-nanocellulose paper.

5. Device characterization optical setup

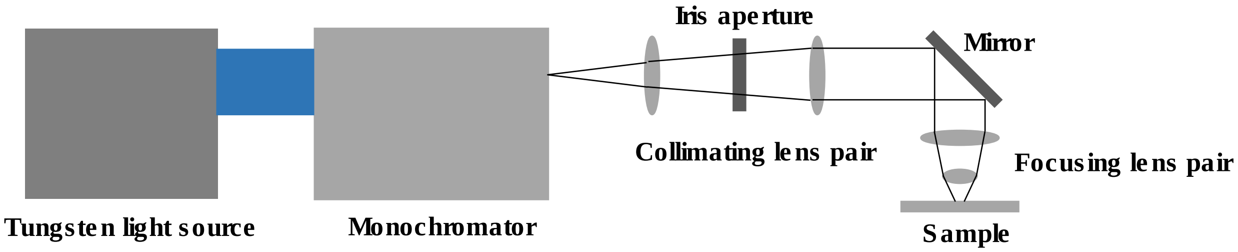

Figure S6 A schematic of the optical setup for photodetector characterization.

Different wavelengths of light are selected through a pair of gratings in the monochromator from a broadband tungsten light source. The diverging light undergoes a collimating lens pair and focuses on the photodetector sample by a focusing lens pair for photocurrent measurements.

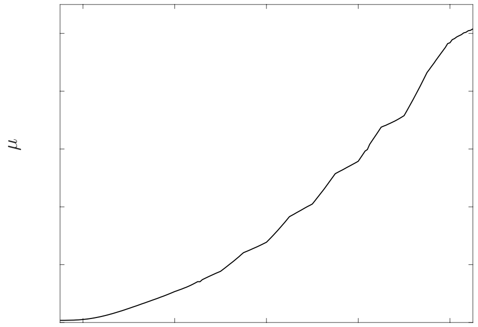

Figure S7 Light power at different wavelengths from the monochromator used for EQE measurement.

References

1. Pedrotti, F.L., Pedrotti, S.J. & Pedrotti, L.S. *Introduction to Optics, 3rd ed. Chapter 7, New Jersey: Prentice Hall* (2007).
